# Case Report: Application of controlled pericardial drainage in type A aortic dissection complicated by cardiac tamponade

**DOI:** 10.3389/fcvm.2025.1595842

**Published:** 2025-08-08

**Authors:** Yixin He, Ying Yang

**Affiliations:** Emergency Department, Peking University Shenzhen Hospital, Shenzhen, Guangdong, China

**Keywords:** controlled pericardial drainage, aortic dissection, cardiac tamponade, bedside echocardiography, computed tomography angiography (CTA)

## Abstract

**Background:**

Type A aortic dissection (TAAD) complicated by cardiac tamponade represents a life-threatening cardiovascular emergency. Acute pericardial effusion can severely compromise cardiac function. Although emergent surgical repair remains the gold standard, preoperative hemodynamic instability substantially increases surgical risk. Controlled pericardial drainage (CPD) has been proposed as a bridging intervention to rapidly relieve tamponade symptoms while mitigating the complications associated with excessive drainage. However, its clinical utility remains controversial because of concerns that the elevation in post-drainage blood pressure may accelerate dissection progression or trigger rupture. This case report highlights the successful use of CPD to stabilize a patient with TAAD and cardiac tamponade, facilitating a safe transition to definitive surgery. It also discusses the clinical utility of this strategy.

**Case presentation:**

A 48-year-old man presented to the emergency department with an acute onset of altered mental status lasting 50 min. Bedside ultrasound and aortic computed tomography angiography confirmed a TAAD diagnosis complicated by pericardial tamponade. After the examination, the patient experienced sudden hypotension, which was promptly managed with emergency CPD, resulting in rapid stabilization of blood pressure. Subsequently, the patient underwent “Sun's procedure” and artificial vascular replacement, during which 500 mL of pericardial blood was extracted. The patient recovered well and was discharged 24 days after the surgery.

**Conclusion:**

With rigorous hemodynamic monitoring and a multidisciplinary framework, CPD effectively functions as a preoperative stabilization approach in patients with TAAD complicated by pericardial tamponade, securing valuable time for subsequent surgical interventions.

## Background

Acute type A aortic dissection (TAAD) is a critical cardiovascular emergency characterized by sudden onset and rapid progression (1–3). If left untreated, mortality increases by 1%–2% per hour within the first 24 h of onset (4). Pericardial tamponade is one of the most severe complications of TAAD (5, 6). The rapid accumulation of hemopericardium leads to cardiac tamponade and exacerbates circulatory collapse, necessitating immediate intervention. Emergency surgery remains the only definitive treatment (7). However, severe preoperative hypotension and hypoperfusion can increase the risk of cardiopulmonary bypass-related complications and postoperative multiorgan failure. In some cases, patients may not even tolerate anesthesia induction.

Controlled pericardial drainage (CPD) has emerged as a potential bridging therapy (8, 9). This technique involves the gradual removal of pericardial fluid via a drainage catheter to alleviate tamponade and improve hemodynamic stability, thereby generating a time window for surgical intervention. CPD is designed to avoid complications from over-drainage. However, the application of CPD remains controversial. Rapid decompression can abruptly elevate blood pressure (BP), subsequently increasing aortic wall shear stress and triggering dissection extension or rupture. Conversely, overly conservative management may delay the procedure beyond the optimal surgical window. Current evidence primarily relies on retrospective studies, and high-quality data on its application and effectiveness in patients with TAAD and cardiac tamponade remain limited.

This article presents a case of TAAD complicated by cardiac tamponade, which was successfully managed with CPD. It elucidates the clinical indications and risk control strategies for CPD in preoperative management, providing insights into clinical decision-making.

## Case presentation

On 2 January 2025, a 48-year-old man presented to the Emergency Rescue Room at Peking University Shenzhen Hospital following an acute episode of altered mental status that had begun 50 min earlier. He had collapsed suddenly with loss of consciousness during a workplace meeting, accompanied by cyanosis but without convulsions or incontinence. Witnesses immediately initiated cardiopulmonary resuscitation (CPR) and called emergency medical services (via the 120 system). Upon arrival, the patient remained unresponsive, and paramedics continued resuscitation. After 5–6 min of CPR, he gradually regained consciousness but remained restless, agitated, and delirious, prompting urgent transfer to the emergency department.

His medical history was significant for cerebral infarction and hypertension. On admission, his vital signs were as follows: temperature 35.5°C, heart rate 112 beats per min, respiratory rate 16 breaths per min, BP 106/64 mmHg, and peripheral oxygen saturation 98%. Physical examination revealed persistent delirium, uncooperative behavior, a sallow complexion, cyanotic lips, and cold, clammy skin. Both pupils were isocoric (2.0 mm diameter) with sluggish light reflexes. Examination of other systems revealed no abnormalities.

**Figure 1:**
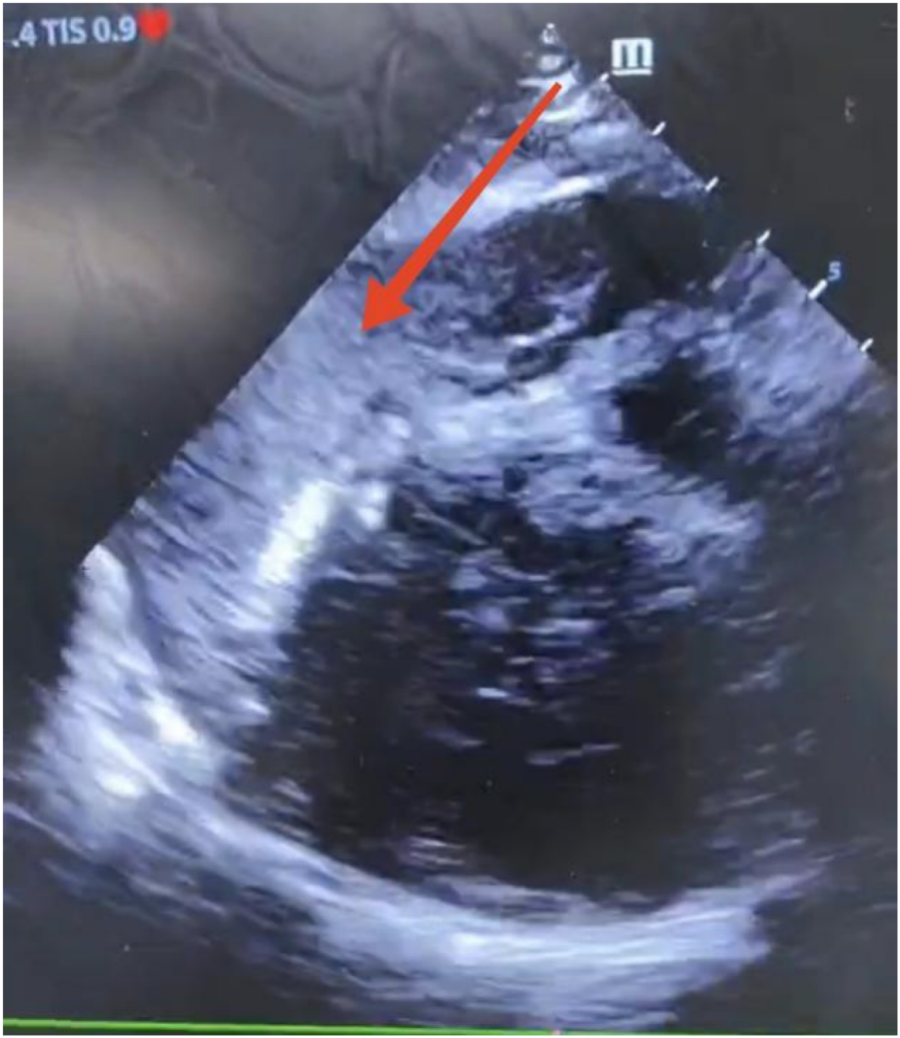
Bedside echocardiography showing a massive pericardial effusion.

**Figure 2:**
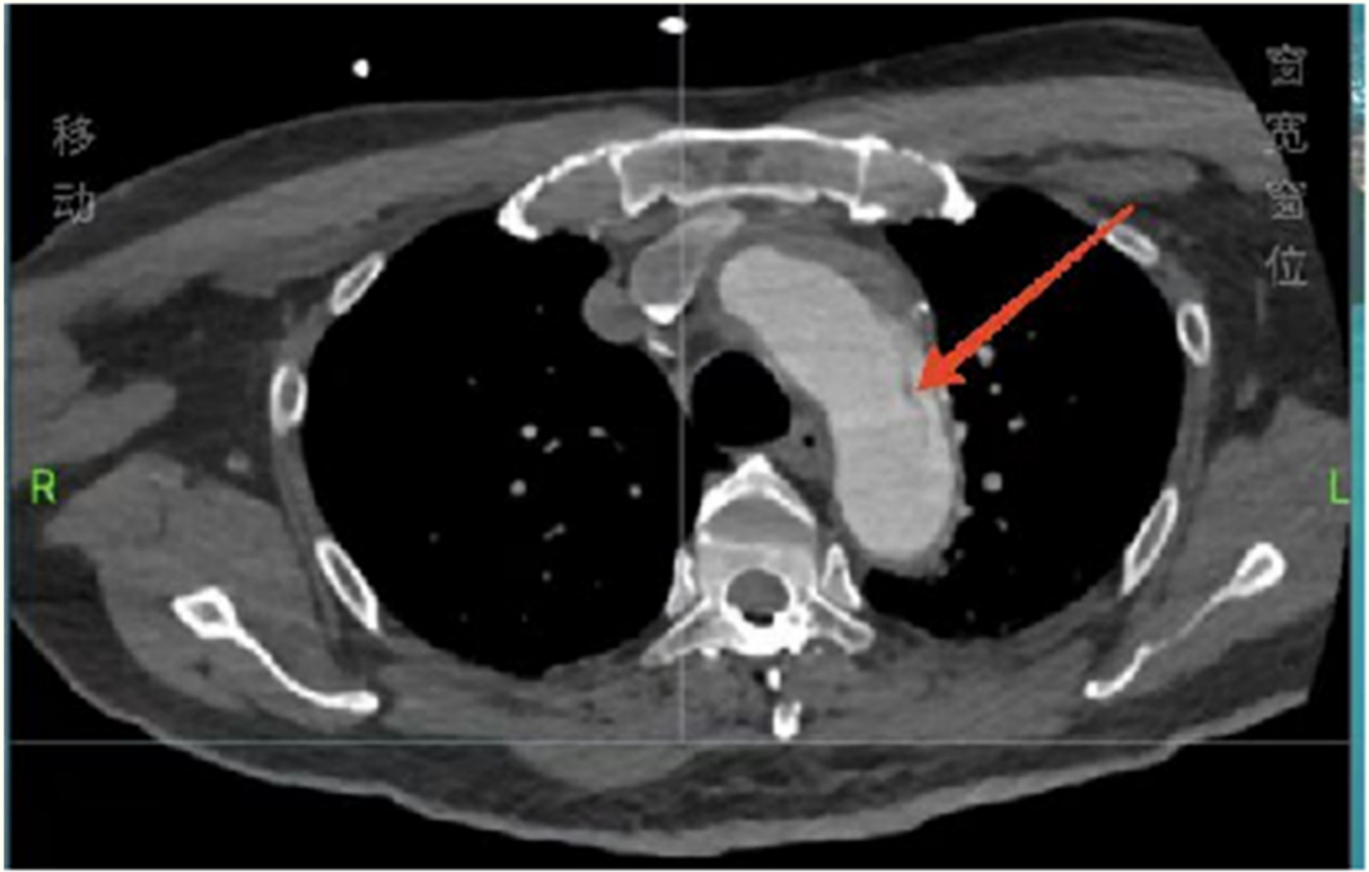
Total aortic computed tomography angiography showing an aortic arch dissection.

**Figure 3:**
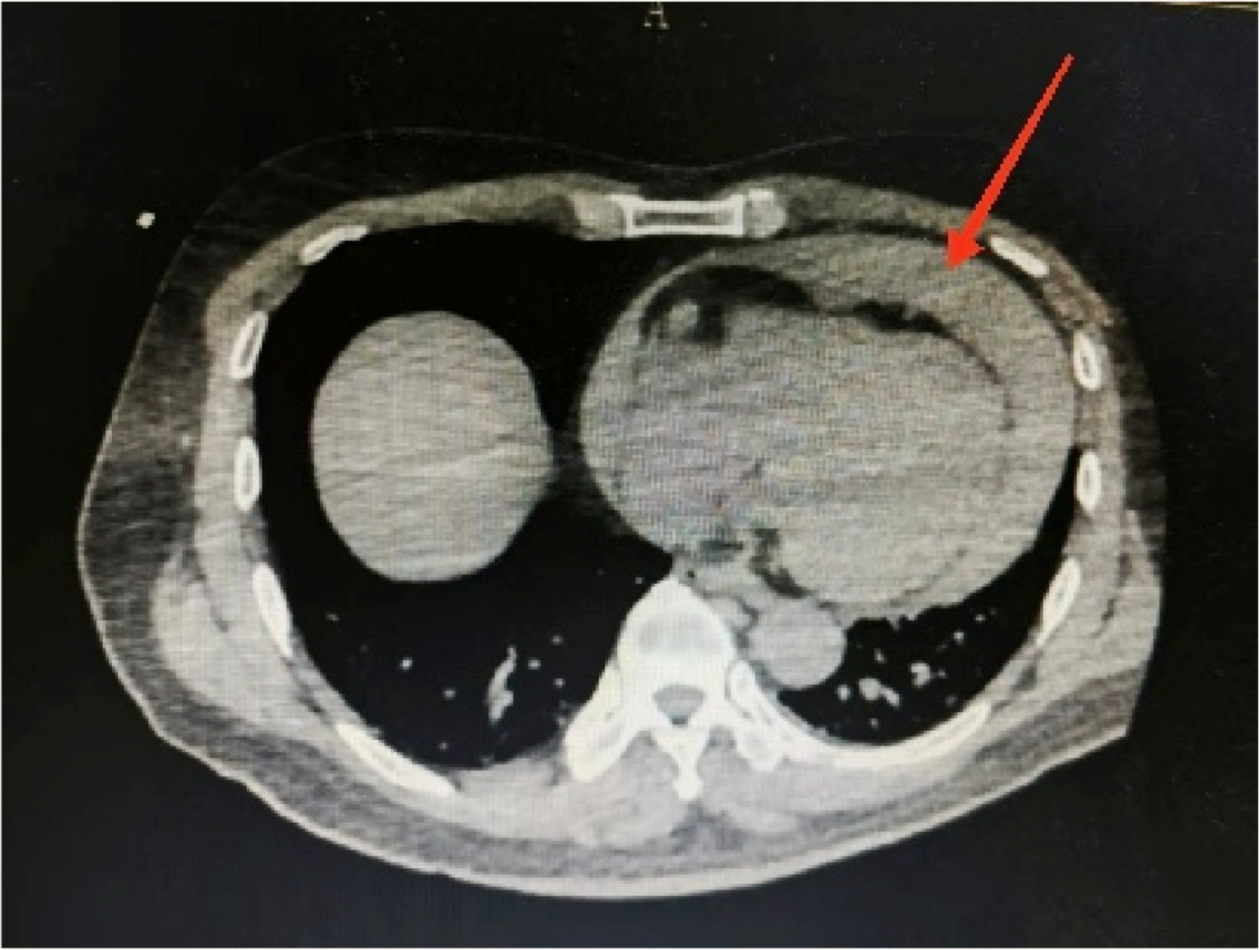
Aortic computed tomography angiography showing a massive hemorrhage of the pericardium.

Considering the patient’s impaired consciousness and uncooperative state, endotracheal intubation and mechanical ventilation were initiated. The initial presentation of cyanosis and cold extremities suggested circulatory shock. Bedside echocardiography, along with laboratory tests, (Table 1) indicated dilation of the ascending aorta and a substantial hypoechoic pericardial effusion (Figure 1), findings highly suggestive of aortic dissection. Once stabilized on a portable ventilator, the patient underwent emergent non-contrast cranial computed tomography (CT) and whole-aortic computed tomography angiography (CTA). Imaging demonstrated hypodense shadows along the aortic arch and anterior abdominal aortic wall, confirming an aortic arch dissection with localized rupture (Figure 2). This resulted in an intramural hematoma extending into the ascending aorta, the aortic arch, and the origin of the left common carotid artery, accompanied by massive pericardial hemorrhage (Figure 3). Cranial CT revealed no abnormalities.

**Table 1:** Laboratory test results.

Laboratory test	Value
WBC (×10^9^/L)	13.03
HGB (g/L)	162
PLT (×10^9^/L)	242
D-D (mg/L FEU)	1.05
cTn I (ng/mL)	0.031
cTn T (ng/mL)	0.019
BNP (pg/mL)	698

WBC, white blood cell; HGB, hemoglobin; PLT, platelet; D-D, d-dimer; cTn I, cardiac troponin I; cTn T, cardiac troponin T; BNP, brain natriuretic peptide.

**Figure 4:**
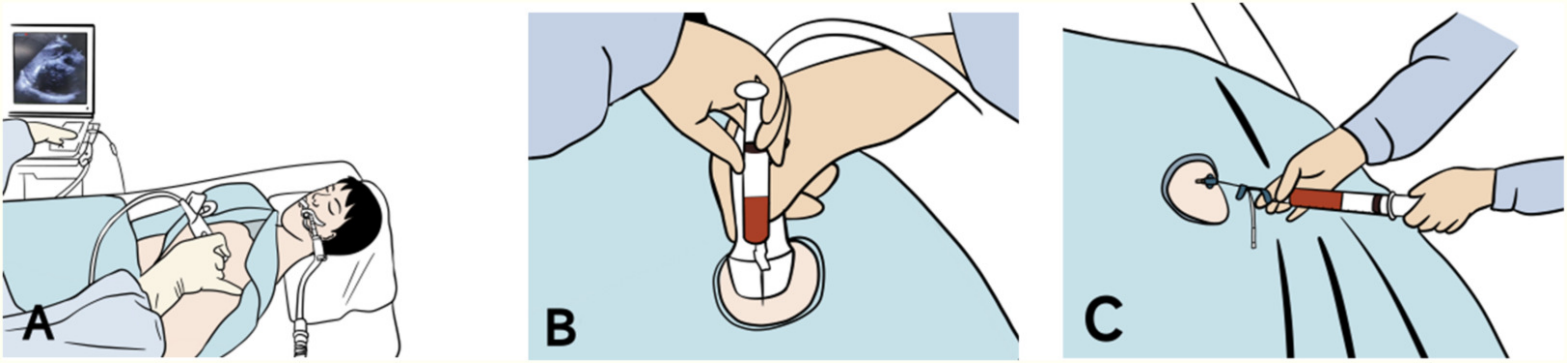
**(A)** Emergency bedside ultrasound showing localization of pericardial effusion. **(B)** First aspiration yields approximately 4 mL of pericardial effusion after needle puncture. **(C)** Second aspiration yields approximately 6 mL of pericardial effusion after successful placement of the drainage catheter.

The patient was returned to the resuscitation room, where he developed hypotension (BP: 64/40 mmHg). Combined imaging and clinical findings suggested a diagnosis of Stanford type A aortic dissection complicated by cardiac tamponade. An emergency bedside pericardial puncture was promptly performed. The patient was placed supine, and local anesthesia was administered. Under ultrasound guidance, the site of maximum pericardial effusion was identified and marked at the fifth intercostal space along the left sternal border (Figure 4). A pericardial puncture was performed at the marked point using an 18-gauge needle inserted perpendicular to the skin. The pericardial space (PS) was accessed within 3.0 cm of the skin. Next, aspiration yielded approximately 4 mL of bloody pericardial effusion. Changes in BP were closely observed to avoid excessive elevation. However, the BP showed no significant improvement (remaining approximately 69/45 mmHg). Consequently, a 7 Fr central venous drainage catheter with multiple side holes (Aspiration Seldinger Kit; Baihe Medical Technology Co., Guangdong, China) was inserted into the PS after the placement of a 0.86-mm guidewire. Drainage volume was carefully controlled through intermittent aspiration using a 10-mL syringe to maintain systolic BP between 90 and 100 mmHg. The patient's blood pressure improved to 108/68 mmHg after an initial aspiration of 6 mL of hemopericardium. Approximately 10 mL of dark crimson, non-coagulating pericardial fluid was extracted. After consulting with the cardiovascular surgery team, the patient's hemodynamic status stabilized, and he was promptly transferred to the cardiac intensive care unit.

**Figure 5:**
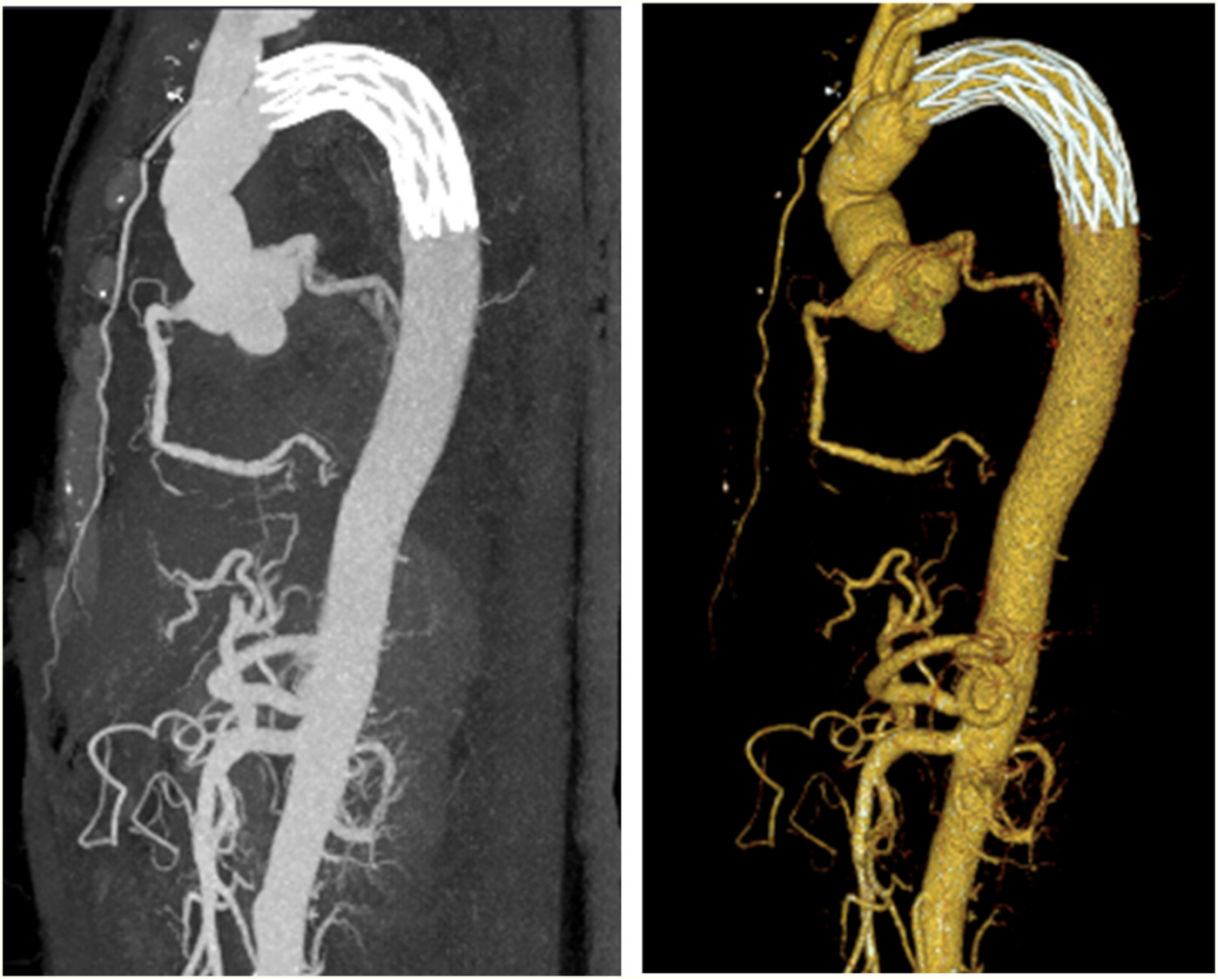
Computed tomography conducted after aortic arch artificial vessel replacement.

Later that day, emergency surgery was conducted using cardiopulmonary bypass under general anesthesia. The procedure is described as follows:

(1) Partial resection of the ascending aorta with artificial vascular graft replacement (Figure 5).
(2) Total aortic arch replacement using a stent-assisted elephant trunk procedure (Sun's procedure).

Intraoperative pericardiotomy suggested approximately 500 mL of hemorrhagic effusion and clots. The patient was transferred to the general cardiac ward on 13 January 2025 and was discharged on 26 January 2025, after a successful recovery. Follow-up continued through 1 June 2025. The Department of Cardiothoracic Surgery conducted follow-up examinations on 27 February, 27 March, 24 April, and 29 May. Cardiac ultrasound and chest x-ray were performed at each visit, and no notable abnormalities were found.

## Discussion

TAAD is the most severe form of aortic dissection, accounting for approximately 60%–70% of all cases. It typically affects individuals between 50 and 70 years of age and is more common in men. The annual incidence is estimated at approximately two to three cases per 100,000 population (10). In untreated cases, mortality increases by approximately 1%–2% per hour within the first 24 h of onset, with an additional 1% increase in mortality risk for each subsequent hour (11). CTA of the aorta remains the most commonly used and diagnostically accurate method, offering high sensitivity and specificity. Surgical intervention remains the gold standard for TAAD treatment, with the primary goal of resecting the affected aortic segment and replacing it with a prosthetic graft to prevent aortic rupture and mitigate the risk of organ ischemia. TAAD complications are often related to the extension of the dissection, impaired blood flow, or aortic rupture. Common complications include myocardial infarction, stroke or cerebral ischemia, renal failure, mesenteric ischemia, and cardiac tamponade. Among these, cardiac tamponade is a life-threatening cardiovascular emergency, associated with an in-hospital mortality rate as high as 54% (12, 13).

In this case, the patient rapidly deteriorated into cardiogenic shock after an acute alteration in consciousness. This condition was a pathophysiological consequence of aortic dissection rupture into the pericardial space, leading to acute cardiac tamponade and subsequent hemodynamic collapse. In such cases, rapid hemodynamic stabilization prior to definitive surgical repair is critical, and the application of controlled pericardial drainage is central to attaining preoperative stability.

Conventionally, pericardial drainage in cases of aortic dissection with tamponade has been approached with caution because of the risk of abrupt pressure reduction, which may exacerbate dissection propagation or cause rupture (14, 15). However, in this hemodynamically unstable patient (BP 64/40 mmHg), delayed intervention would have inevitably resulted in circulatory collapse. In this case, ultrasound-guided CPD allowed drainage of only 10 mL of hemorrhagic fluid; however, it promptly restored BP to 108/68 mmHg. This observation aligns with the steep pressure–volume relationship characteristic of acute cardiac tamponade, in which rapid fluid accumulation leads to disproportionate increases in intrapericardial pressure, whereas minimal early decompression can result in significant hemodynamic improvement (16, 17). The controlled approach strikes a balance between the risk of catastrophic rebleeding and the urgent need to reverse circulatory failure.

This finding further supports the results reported by Hayashi et al., who observed that patients undergoing pericardiocentesis for tamponade had an average drainage volume of 40.1 + 30.6 mL, with 10 patients requiring drainage of only ≤30 mL (18). The present case further supports the notion that even a small drainage volume (10 mL) can effectively relieve pericardial pressure and significantly improve hemodynamic status. This finding not only supplements the data from Hayashi et al. but also supports the clinical utility of a customized, minimal-volume drainage strategy in emergency settings, specifically in the treatment of hemodynamically unstable patients who are at risk of aortic rupture.

Furthermore, this case exemplifies the “damage control resuscitation” strategy. Controlled drainage minimized procedural risks (e.g., avoiding excessive decompression that could trigger dissection extension) while creating a hemodynamically stable window that facilitated safe progression to Sun's procedure—a total arch replacement using the stented elephant trunk technique. Notably, the marked discrepancy between the intraoperative pericardial clot volume (500 mL) and the preoperative drainage volume (10 mL) suggests intermittent hemorrhage from the dissection aorta. CPD may have mitigated the risk of further rupture by maintaining hemodynamic equilibrium between intrapericardial and aortic luminal pressures, thereby preventing abrupt decompression. Hiratzka et al.'s “pressure equilibrium theory” is in line with this finding (19).

This case presents unique insights into the clinical management of TAAD complicated by pericardial tamponade and is notable in several respects. First, the controlled pericardial drainage strategy differs from traditional methods that typically involve pericardial evacuation in large volumes. To maintain systolic BP within a safe range (approximately 90–100 mmHg), the clinical team relied on bedside, ultrasound-guided pericardial puncture with intermittent, low-volume aspiration that was carefully adjusted. This strategy avoided the sudden elevation in BP that may occur with a single large-volume drainage, thereby reducing the risk of triggering rupture. In this case, the drainage of only 10 mL of hemorrhagic pericardial effusion immediately restored BP to 108/68 mmHg, indicating that minimal fluid drainage can effectively relieve pericardial pressure. This finding provides an important reference for managing similar cases.

Second, this case involved a highly unstable patient who presented with a critically low BP of 64/40 mmHg, was intubated, and exhibited typical signs of pericardial tamponade, such as cyanosis, cold and clammy skin, and altered mental status. In this emergency setting, prompt decision-making and CPD implementation created a vital therapeutic window for subsequent emergency surgery.

Third, multidisciplinary collaboration played a crucial role in the successful management of this case. Upon admission, the emergency department quickly initiated its response protocol and immediately coordinated with the ultrasound team to perform bedside echocardiography, which confirmed the presence and severity of the pericardial effusion. This imaging modality provided essential evidence for subsequent interventions. Simultaneously, we promptly contacted the radiology department to conduct an aortic CTA to further assess the aortic condition and guide surgical planning. With the support of the emergency department, endotracheal intubation and CPD were successfully performed, stabilizing the patient's vital signs. The patient was then urgently transferred to the cardiothoracic surgery department for further treatment. The relevance of collaborative care in managing complicated cardiovascular emergencies is demonstrated by this meticulously planned, multidisciplinary workflow. The seamless integration of services not only improved the patient's survival but also provided a clinical paradigm for the management of similar cases in the future. In summary, this case provides new insights and practical experience for the clinical management of TAAD complicated by pericardial tamponade: it highlights the feasibility and safety of CPD, its application in high-risk patients, and the importance of multidisciplinary collaboration.

Several crucial elements must be considered when implementing CPD, and its application should be customized for each individual clinical situation. First, the patient’s hemodynamic status is a critical determinant in guiding the decision to initiate or continue drainage. For instance, in this case, hypoperfusion symptoms, such as altered mental status, hypotension, and cold, clammy skin, indicated the urgent need for decompression. Second, the safety and effectiveness of procedures depend on real-time imaging. The rapid identification of pericardial effusion and aortic dilatation by point-of-care ultrasound, followed by CTA to delineate the dissection location, facilitates precise needle placement and informed drainage planning. In addition, multidisciplinary collaboration is crucial for procedural success. Real-time coordination among the emergency department, ultrasound department, cardiac surgery, and radiology ensures a seamless transition from diagnosis to intervention.

However, this technique carries certain risks. For example, complications, such as coronary artery injury, secondary dissection extension, and recurrent pericardial effusion, should be carefully monitored. Moreover, the optimal drainage volume remains undefined, and further research is warranted to develop personalized drainage strategies based on real-time hemodynamic monitoring.

The European Society of Cardiology guidelines do not provide specific recommendations regarding the use of CPD in TAAD complicated by pericardial tamponade (20). Nevertheless, case reports suggest that CPD may serve as a temporizing measure under strict conditions, such as the inability to proceed with immediate surgery and the use of ultrasound guidance for puncture (21, 22). The successful outcome of this case highlights the potential of CPD to stabilize hemodynamic status and extend the surgical window for subsequent intervention in selected patients.

Future research should focus on the following areas:

(1) Individualized drainage strategies, using hemodynamic monitoring devices (e.g., pericardial pressure sensors), to optimize drainage protocols, determine the minimum effective drainage volumes, and define optimal drainage rates.
(2) Long-term outcome studies to evaluate the impact of CPD on postoperative recovery and long-term survival.
(3) Standardization of procedural guidelines and training to improve technical proficiency in emergency settings.
(4) Promotion of multidisciplinary collaboration models to establish a replicable framework for the management of similar complex cardiovascular emergencies.

## Data Availability

The original contributions presented in the study are included in the article/Supplementary Material, further inquiries can be directed to the corresponding author.
